# An interaction between *Nrf2 *polymorphisms and smoking status affects annual decline in FEV_1_: a longitudinal retrospective cohort study

**DOI:** 10.1186/1471-2350-12-97

**Published:** 2011-07-20

**Authors:** Hironori Masuko, Tohru Sakamoto, Yoshiko Kaneko, Hiroaki Iijima, Takashi Naito, Emiko Noguchi, Tomomitsu Hirota, Mayumi Tamari, Nobuyuki Hizawa

**Affiliations:** 1 Division of Respiratory Medicine, Institute of Clinical Medicine, University of Tsukuba, Ibaraki, Japan; 2 Tsukuba Medical Center, Ibaraki, Japan; 3 Department of Medical Genetics, Majors of Medical Sciences, Graduate School of Comprehensive Human Sciences, University of Tsukuba, Ibaraki, Japan; 4 Laboratory for Respiratory Diseases, Center for Genomic Medicine, the Institute of Physical and Chemical Research (RIKEN), Kanagawa, Japan

## Abstract

**Background:**

An Nrf2-dependent response is a central protective mechanism against oxidative stress. We propose that particular genetic variants of the *Nrf2 *gene may be associated with a rapid forced expiratory volume in one second (FEV_1_) decline induced by cigarette smoking.

**Methods:**

We conducted a retrospective cohort study of 915 Japanese from a general population. Values of annual decline in FEV_1 _were computed for each individual using a linear mixed-effect model. Multiple clinical characteristics were assessed to identify associations with annual FEV_1 _decline. Tag single-nucleotide polymorphisms (SNPs) in the *Nrf2 *gene (rs2001350, rs6726395, rs1962142, rs2364722) and one functional SNP (rs6721961) in the *Nrf2 *promoter region were genotyped to assess interactions between the *Nrf2 *polymorphisms and smoking status on annual FEV_1 _decline.

**Results:**

Annual FEV_1 _decline was associated with smoking behavior and inversely correlated with FEV_1_/FVC and FEV_1 _% predicted. The mean annual FEV_1 _declines in individuals with rs6726395 G/G, G/A, or A/A were 26.2, 22.3, and 20.8 mL/year, respectively, and differences in these means were statistically significant (p_corr _= 0.016). We also found a significant interaction between rs6726395 genotype and smoking status on the FEV_1 _decline (p for interaction = 0.011). The haplotype rs2001350T/rs6726395A/rs1962142A/rs2364722A/rs6721961T was associated with lower annual decline in FEV_1 _(p = 0.004).

**Conclusions:**

This study indicated that an Nrf2-dependent response to exogenous stimuli may affect annual FEV_1 _decline in the general population. It appears that the genetic influence of *Nrf2 *is modified by smoking status, suggesting the presence of a gene-environment interaction in accelerated decline in FEV_1_.

## Background

Among pulmonary function test (PFT) measurements, forced expiratory volume in one second (FEV_1_) is the most reproducible [1]. Therefore, it is suitable for analyzing changes in pulmonary function over time. Accelerated decline in FEV_1 _is considered as an important predictor for the development of inflammatory obstructive lung diseases, such as asthma and chronic obstructive pulmonary disease (COPD) [2,3]. A rapid decline in FEV_1 _may be affected by multiple factors, including environmental and genetic factors.

The most important environmental factor for FEV_1 _decline is cigarette smoking. In their landmark study, Fletcher et al. [4] demonstrated that smokers had a steeper decline in FEV_1 _than non-smokers. Subsequent studies have revealed that the rate of decline in FEV_1 _depends on pack-years smoked and that the accelerated decline in FEV_1 _in smokers slows to normal rates of decline upon smoking cessation [4-6]. Cigarette smoke contains high concentrations of oxidants, including reactive oxygen species and reactive nitrogen species [7]. Oxidative stress due to cigarette smoking promotes direct injury to airway epithelium, expression of genes encoding proinflammatory mediators, and protease/antiprotease imbalance [8], all of which induce chronic inflammation in the lung of smokers that results in deterioration of lung function.

However, only 10-15% of smokers develop a severe impairment of lung function [4]. In addition to environmental factors, genetic determinants play an important role in rapid decline in lung function. A pedigree-based study has shown that FEV_1 _levels have a heritability that is independent of cigarette smoking and disease status such as asthma [9]. Furthermore, recent large-scale genome-wide association studies have identified several loci associated with FEV_1 _and the FEV_1_/forced vital capacity (FVC) ratio [10,11].

It is possible that oxidant/antioxidant imbalance in the lungs of smokers results in an accelerated loss of lung function. Nrf2 is a major regulator of the antioxidant response [12]. Nrf2 regulates the expression of several genes encoding antioxidant and detoxification proteins [13]. In animal models, Nrf2 plays an important role in reducing inflammation associated with elastase-induced emphysema [14]. In human studies, attenuation of Nrf2 due to the down-regulation of the *Nrf2 *mRNA has been detected in alveolar macrophages of COPD patients [15]. Moreover, 3 single-nucleotide polymorphisms (SNPs) in the promoter region of the *Nrf2 *gene have an influence on the gene's transcriptional activity, and one of these SNPs is associated with the development of acute lung injury [16]. Recently, one SNP (rs2364723) in the first intron of *Nrf2 *has been shown to be related to a lower FEV_1 _[17]. All of these findings indicate that an Nrf2-dependent adaptive response is important in inhibiting the oxidant-induced lung inflammation that results in a rapid decline in lung function.

Therefore, we conducted a longitudinal retrospective cohort study of a general Japanese population in order to analyze associations between *Nrf2 *polymorphisms and annual decline in FEV_1_. We also assessed whether an interaction between the *Nrf2 *polymorphisms and smoking status affects FEV_1 _decline.

## Methods

### Subjects

**Figure 1:**
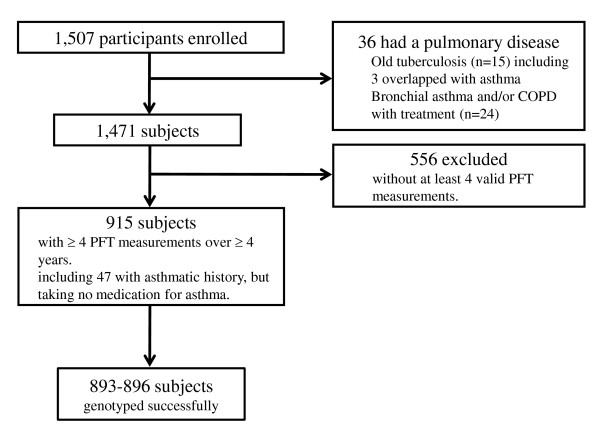
**Schematic representation of the study profile**.

A retrospective cohort study was conducted. We recruited 1,507 full-blooded Japanese subjects from a general population who visited the Tsukuba Medical Center for annual health checkup from June 2008 to May 2009 (Figure 1). Detailed information on the cohort is available in a previous report [18]. The individuals completed questionnaires concerning respiratory health, medical history, lifestyle, and exposure to environmental irritants (e.g. cigarette smoke, allergens, and air pollution). All 1,507 subjects participated in a medical interview, a physical examination, routine blood studies, a chest roentgenogram, and PFTs. Based on detailed data from these analyses, we excluded 36 subjects from the study because of a preexisting lung ailment; 12 were diagnosed as having preexisting tuberculosis, 3 also had a diagnosis of asthma, and 24 individuals with asthma or COPD who had been treated by inhaled corticosteroids, leukotriene receptor antagonists, and/or bronchodilators such as β-adrenoceptor stimulants and anticholinergic agents. From among the remaining 1,471 subjects, 915 participants who took at least 4 valid PFT measurements over a period of at least 4 years were selected for this study in order to ensure a reliable estimate of longitudinal decline in FEV_1_. The final study population of 915 participants included 47 subjects with asthmatic history who had not taken asthma medication during the retrospective study period.

The Institutional Review Boards of the University of Tsukuba (IRB No. 136) and the Tsukuba Medical Center (IRB No. 2008-01-31) approved the study, and each subject provided written informed consent.

### Pulmonary Function Test (PFT)

Spirometry was performed with an electronic spirometer (Autospiro SYSTEM7; Minato Medical Science Co., Ltd., Osaka, Japan) according to the standards recommended by the Japanese Respiratory Society (JRS) [19]. The patients performed the maneuvers without any bronchodilators. The highest value for the sum of FVC and FEV_1 _was selected as the measurement for each PFT. FVC and FEV_1 _were expressed as a percentage of predicted values approved by the JRS [19]. All available longitudinal data for each participant were collected retrospectively to estimate the annual decline in FEV_1_.

### Single-Nucleotide Polymorphism (SNP) Selection and Genotyping

Using JPT (Japanese in Tokyo, Japan) genotype data (PhaseIII/Rel#2, Feb09, on NCBI B36 assembly, dbSNP b126) from the International HapMap project http://hapmap.org/, four tag SNPs (rs2001350, rs6726395, rs1962142, rs2364722) were identified in the 34.38 kb *Nrf2 *gene region (chromosome 2, position 177,803,285-177,837,663). We used the multi-marker predictor method implemented in the Tagger program [20]. Tag set was generated using a threshold r^2 ^of 0.8 and a minor allele frequency of > 0.1. Genomic DNA was extracted from samples of whole blood from each of the 915 participants by an automated DNA extraction system (QuickGene-610L, FUJIFILM, Tokyo, Japan). Genotyping of these 4 bi-allelic SNPs were attempted for each participant by the pre-designed TaqMan allele-specific polymerase chain reaction (PCR) assays according to the manufacturer's instruction (Applied Biosystems, Foster City, CA).

It has been reported that 3 SNPs (rs6721961, rs6706649, and rs35652124) located in the promoter region of the *Nrf2 *gene affect the transcriptional activity of *Nrf2 *[16]. Genotyping for rs6721961 was carried out for each participant by the TaqMan technique using a pair of primers and a pair of oligonucleotide probes designed and synthesized by Applied Biosystems. The sequences of the primers were as follows: forward, 5'-CAGTGGGCCCTGCCTAG-3'; reverse, 5'-TCAGGGTGACTGCGAACAC-3'. The TaqMan fluorescence-labeled oligonucleotide probes were 5'-[VIC]-TGGACAGCGCCGGCAG-3' and 5'-[FAM]-TGTGGACAGCTCCGGCAG-3'. Because rs6706649 and rs35652124 are only 2 base pairs apart, the allele-specific probe technique was not appropriate for genotyping. Instead, for these 2 SNPs, direct DNA sequencing analysis was performed for 50 subjects (25 major allele homozygotes and 25 minor allele homozygotes for rs6726395 SNP). PCR amplification was carried out with 50 ng genomic DNA and a pair of primers flanking the 2 SNPs by a GeneAmp PCR System (Applied Biosystems). The primer sequences were as follows: forward, 5'-AGAGGTTCTCTTGGGGTTCC-3'; reverse, 5'-AGAACCTTGCCCTGCTTTTA-3'. The amplified 343-bp PCR DNA products were sequenced using the same primers and the dideoxynucleotide chain termination method available as a fluorescent sequencing kit (DNA Sequencing Kit; Applied Biosystems) and an automated sequencer (ABI PRISM 3130; Applied Biosystems) according to the manufacturers' instruction.

### Statistics

Data are expressed as mean ± SD, unless otherwise stated. Statistical analysis was performed using SYSTAT software, version 13 (Systat Software, Inc., Chicago, IL). Statistical tests with a p value < 0.05 were considered significant.

Values of annual FEV_1 _decline were computed for each individual across the repeated measurements using a linear mixed-effect model. We used a random intercept to take into account the heterogeneity across subjects and the correlation induced by having repeated observations on the same subjects.

We performed univariate analysis to evaluate association of annual decline in FEV_1 _with clinical characteristics. For categorical variables such as gender and smoking status, Student's *t *tests and one-way analyses of variance with Bonferroni *post hoc *correction were used for comparisons of 2 and 3 group means, respectively. For continuous variables such as age, body mass index (BMI), PFT measurements, and total serum IgE levels, the correlation with annual decline in FEV_1 _was assessed by Pearson correlation coefficient analysis.

All polymorphisms were tested for Hardy-Weinberg equilibrium using Haploview 4.2 software http://www.broadinstitute.org/haploview[21]. Estimates of pairwise linkage disequilibrium (LD) between the loci were calculated using r^2 ^[22]. The associations of genotypes with annual decline in FEV_1 _were analyzed by multivariate linear regressions adjusted for potential confounding factors such as sex, age, BMI, FEV_1_/FVC ratio, total serum IgE levels, smoking status (never, ex, or current), smoking index (0, 0-200 or > 200), and affection of bronchial asthma. Correction for multiple comparisons was done by the Bonferroni's method. The interaction effect of genotypes and smoking status on the annual decline in FEV_1 _was analyzed using general linear models adjusted for the same confounding factors except for smoking behavior.

Association of the rs6726395 genotypes with the mRNA expression levels of *Nrf2 *was analyzed using GENEVAR database http://www.sanger.ac.uk/humgen/genevar/[23], which shows mRNA expression profiles of 3 cell types (fibroblast, lymphoblastoid cell line and T-cell) derived from umbilical cords of 75 Geneva GenCord individuals [24].

For analyses of association between haplotypes and annual FEV_1 _decline, we used the Haplo. score program http://mayoresearch.mayo.edu/mayo/research/biostat/schaid.cfm, which adjusts for the same covariates and calculates simulation p values for each haplotype [25].

## Results

Characteristics of the study cohort, 915 Japanese individuals from a general population, are provided in Table 1. The average number of visits for routine health checkups over the study period per participant was 9.1 ± 3.7 times during 11.1 ± 4.6 years. The mean age at the recruitment was 52.1 years (31-78 years). All the participants were over 25 years of age at the first visit. Of the participants, 63% were never-smokers, 23% were ex-smokers, and 14% were current-smokers. The average of serum IgE levels was 1.78 (log IU/mL) (normal, < 2.23). The mean annual decline in FEV_1 _was 23.8 mL/year. The final study population included 47 subjects with a history of asthma, but each of these subjects had not been treated with asthma medications.

**Table 1:** Characteristics of the participants^a^

Characteristic	Participants (N = 915)
Age (years)	52.1 ± 8.3
Male sex - N (%)	418 (45.7)
Total duration of follow-up (years)	11.1 ± 4.6
Number of visits	9.1 ± 3.7
BMI	23.1 ± 3.0
FVC (L)	3.12 ± 0.78
FVC % predicted (%)	100.5 ± 13.6
FEV_1 _(L)	2.56 ± 0.64
FEV_1_/FVC (%)	82.2 ± 5.6
FEV_1 _decline (mL/year)	23.8 ± 26.3
Serum IgE (Log IU/mL)	1.78 ± 0.59
Smoking habits - N (%)	
Never-smokers	576 (63.0)
Ex-smokers	210 (23.0)
Current-smokers	129 (14.1)
Smoking index^b ^- N (%)	
0	576 (63.0)
0-200	114 (12.5)
> 200	225 (24.6)
Asthma - N (%)	47 (5.1)

^a ^Data are presented as mean ± SD or n (%).

^b ^Smoking index was calculated for current and past smokers by multiplying smoking dose (cigarettes per day) and duration (years smoked).

Univariate analysis was performed to evaluate relationships between annual decline in FEV_1 _and clinical variables (Table 2). There was no significant difference in FEV_1 _decline between men and women. As expected, the association between annual FEV_1 _decline and smoking behavior was statistically significant. The mean value of annual FEV_1 _declines in current-smokers was significantly larger than that in never-smokers. We calculated smoking index (cigarettes/day × years smoked) for all the participants and divided the study population into 3 categories; 0, 0-200, and > 200. Mean FEV_1 _declines in the 0-200 and > 200 groups were significantly larger than that in the 0 group. The value of FEV_1 _decline showed a weak inverse correlation with FEV_1_/FVC (r = -0.278, p < 0.001) and FEV_1 _% predicted (r = -0.263, p < 0.001). As for the pairwise correlation between annual FEV_1 _decline and the other pulmonary function measurements, the correlation coefficients were -0.146 for FVC % predicted (p < 0.001), -0.144 for FEV_1 _(p < 0.001), and -0.069 for FVC (p = 0.037). The correlation coefficients for annual FEV_1 _decline and age was 0.105 (p = 0.001) and that for annual FEV_1 _decline and total IgE levels was 0.097 (p = 0.003).

**Table 2:** Univariate analysis comparing annual decline in FEV_1 _and clinical variables

Variables	Correlation coefficient^a^	Annual decline in FEV_1 _ Mean ± SD (mL/yr)	P value
Categorical variables			
Gender			0.205
Male		25.0 ± 28.2	
Female		22.8 ± 24.5	
Smoking habits			0.005
Never-smokers		21.7 ± 24.5	
Ex-smokers		26.7 ± 27.6	
Current-smokers		28.5 ± .30.5*	
Smoking index^b^			0.006
0		21.7 ± 24.5	
0-200		28.2 ± 29.6	
> 200		27.0 ± 28.3*	
Continuous variables			
Age	0.105		0.001
BMI	0.038		0.245
FVC (L)	-0.069		0.037
FVC % predicted (%)	-0.146		< 0.001
FEV_1 _(L)	-0.144		< 0.001
FEV_1 _% predicted (%)	-0.263		< 0.001
FEV_1_/FVC (%)	-0.278		< 0.001
Serum IgE (Log IU/mL)	0.097		0.003

^a ^Pearson coefficient.

^b ^Smoking index was calculated for current and past smokers by multiplying smoking dose (cigarettes per day) and duration (years smoked).

* p < 0.05 compared with never-smokes (smoking index 0) after Bonferroni *post hoc *correction.

Genotype analysis at 4 tag SNPs (rs2001350, rs6726395, rs1962142, rs2364722) and one previously-reported functional SNP (rs6721961) [16] in the *Nrf2 *promoter region was attempted for the each participant. Genotyping of rs2001350 and rs6726395 was successful for 895 subjects; similarly, rs1962142 and rs2364722 genotyping was successful for 896 subjects, and rs6721961 was for 893 subjects. The overall success rate was 97.6-97.9%. All the analyzed SNPs were in Hardy-Weinberg equilibrium. Relationships between the genotypes and the annual FEV_1 _decline are shown in Table 3. Annual FEV_1 _declines adjusted for potential confounding factors were significantly different among rs6726395 genotypes (p_corr _= 0.016). The mean annual FEV_1 _declines in major allele homozygotes (G/G), heterozygotes (G/A), and minor allele homozygotes (A/A) for the rs6726395 SNP were 26.2, 22.3, and 20.8 mL/year, respectively. In contrast, the GENEVAR database did not show a significant difference in *Nrf2 *mRNA expression levels based on rs6726395 genotypes (data not shown).

**Table 3:** Multivariate linear regressions^a ^for association between genotypes at the *Nrf2 *SNPs and decline in FEV_1_

SNP	Genotype	N	Annual decline in FEV_1_ Mean ± SD (mL/year)	P value (corrected p value)
rs2001350	CC	43	24.7 ± 7.5	0.101
	CT	296	24.2 ± 7.4	
	TT	551	23.8 ± 7.9	
rs6726395	GG	431	26.2 ± 8.3	0.0031 (0.016)
	GA	378	22.3 ± 7.4	
	AA	86	20.8 ± 8.8	
rs1962142	AA	23	28.1 ± 10.4	0.194
	AG	277	23.6 ± 6.8	
	GG	591	24.0 ± 8.0	
rs2364722	AA	157	24.9 ± 7.9	0.277
	AG	421	24.0 ± 7.4	
	GG	313	23.4 ± 8.1	
rs6721961	GG	496	23.6 ± 7.8	0.900
	GT	344	24.5 ± 7.7	
	TT	53	24.4 ± 7.3	

^a ^Adjusted for sex, age, BMI, FEV_1_/FVC ratio, serum IgE levels, smoking status, smoking index, and asthma diagnosis.

**Figure 2:**
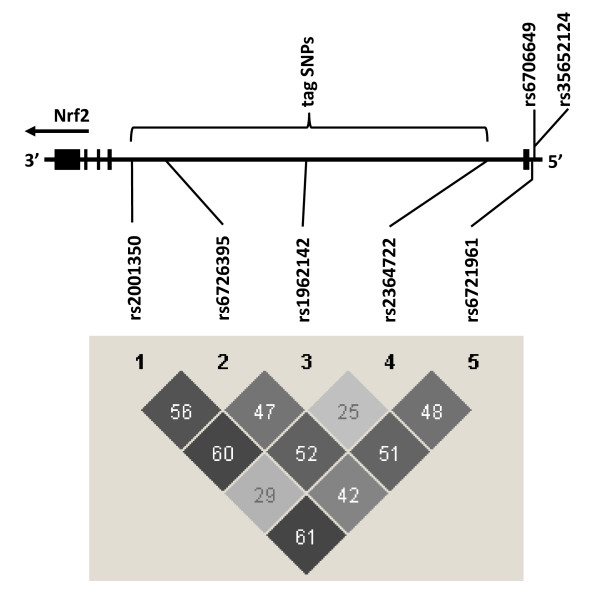
**Genomic organization of the *Nrf2 *gene and linkage disequilibrium (LD) map for single-nucleotide polymorphisms (SNPs)**. Exons are shown as black boxes. Note that the reference sequence uses the complementary strand of DNA; thus, the 5' region is on the right. The 4 tag SNPs are located in the first intron and the 3 functional SNPs are in the promoter region. Pairwise LD values (r^2 ^× 100) for the 4 tag SNPs and rs6721961 were calculated based on genotypes of the whole study population. The intensity of the gray shading in squares of the LD map is proportional to r^2^.

Pairwise LD (r^2^) values among the 5 SNPs are shown in Figure 2. In the present study, the observed LD values among the 4 tag SNPs detected in the HapMap project corresponded well with the LD structure that is expected based on the patterns in the JPT (Japanese) population of the HapMap group. All the r^2 ^values among the 5 SNPs studied did not exceed 0.61, indicating that these 5 SNPs were not in tight LD with each other.

Previous results indicate that 3 SNPs (rs6721961, rs6706649 and rs35652124) in the promoter region of *Nrf2 *are functionally relevant [16]. Although it has been reported that the minor allele of rs6721961 SNP diminishes promoter activity of the *Nrf2 *gene, there was no significant association between this SNP and annual FEV_1 _decline in the present study (Table 3). We estimated the extent of linkage disequilibrium between rs6726395 and the other 2 functional SNPs (rs6706649 and rs35652124) by sequencing 50 subjects. The SNP rs6726395 was not in tight LD with rs6706649 (r^2 ^= 0.02) or with rs35652124 (r^2 ^= 0.67), suggesting that genetic effects of these 2 functional SNPs do not underlie the association of rs6726395 with FEV_1 _decline. The SNP rs35652124 was in tight LD (r^2 ^= 0.92) with rs2364722, which did not have a significant association with annual decline in FEV_1 _in the present study (Table 3).

**Figure 3:**
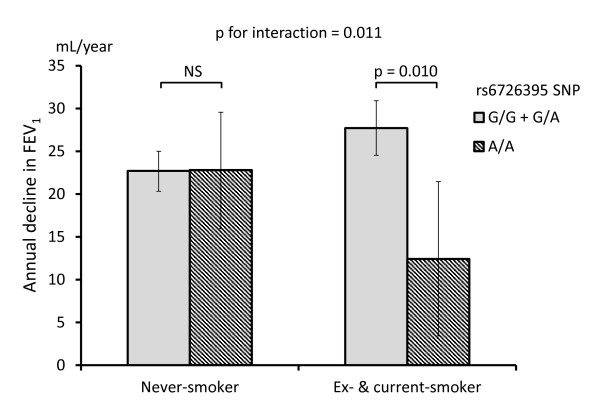
**Effect of interaction between rs6726395 SNP and cigarette smoking status on annual decline in FEV_1_**. Data are presented as mean (95% CI). Light gray bar and striped bar represent rs6726395 G/G + G/A and A/A genotype groups, respectively. Only in ex- and current-smokers was the annual FEV_1_ decline significantly different between the 2 groups. The interaction effect was analyzed by general linear models adjusted for sex, age, BMI, FEV_1_/FVC ratio, serum IgE levels, and affection of bronchial asthma.

Next, we analyzed effect of interaction between the bi-allelic SNP rs6726395 and cigarette smoking status on annual decline in FEV_1 _(Figure 3). In ex- and current-smokers, annual FEV_1 _decline was significantly different between individuals with and without the rs6726395 G allele for FEV_1 _decline. The mean decline values were 27.8 mL/year and 12.5 mL/year in individuals with the G/G + G/A genotype and those with the A/A genotype, respectively (p = 0.010). In contrast, the annual FEV_1 _decline was not significantly different between individuals carrying the G/G + G/A and the A/A genotypes (22.7 and 22.8 mL/year, respectively) for individuals who were never-smokers. The p value for interaction between rs6726395 and smoking status on the annual decline in FEV_1 _was 0.011. These results indicated that the genetic influence of *Nrf2 *was modified by smoking status, suggesting the presence of a gene-environment interaction in annual decline in FEV_1_.

We constructed haplotypes composed of the 4 tag SNPs and rs6721961, and analyzed association of the haplotypes with annual FEV_1 _decline (Table 4). The distribution of the haplotypes was significantly related to annual FEV_1 _decline with the global simulation p value of 0.004. We identified 4 common haplotypes covering 86.8% of the whole genotyped population. The haplotype most strongly associated with annual FEV_1 _decline was rs2001350T/rs6726395A/rs1962142A/rs2364722A/rs6721961T with a haplotype score of -2.988 and a simulation p value of 0.002; this haplotype was associated with lower decline in FEV_1_.

**Table 4:** Estimated haplotype frequencies and haplotype association with annual decline in FEV_1_

Haplotype	rs2001350	rs6726395	rs1962142	rs2364722	rs6721961	Haplotype frequency	Haplotype-specific score	Simulation p value	Global simulation p value
1	T	A	A	A	T	0.082	-2.988	0.002	0.004
2	C	G	A	G	T	0.148	-1.319	0.182	
3	T	A	G	A	G	0.561	0.453	0.663	
4	T	A	A	A	G	0.077	1.184	0.235	

Haplotype frequencies were estimated using the Haplo. Stats program. Haplotype 1(rs2001350T/rs6726395A/rs1962142A/rs2364722A/rs6721961T) was associated with annual decline in FEV_1 _with a haplotype score of -2.988 and a simulation p value of 0.002. Haplotye with frequencies less than 0.05 were excluded.

## Discussion

Cigarette smoke (CS) contains a high concentration of oxidants and leads to oxidative stress [7]. In smokers, increased oxidative stress in the airways is a predominant cause of accelerated decline in lung function [26,27]. Nrf2 plays a central role in protecting the lung against CS-induced oxidative stress by up-regulating multiple genes encoding antioxidant and detoxification proteins, e.g., heme oxygenase-1, NADPH, and glutathione S-transferase [12,13]. CS-induced reactive oxygen species production via NADPH oxidase activation is involved in the positive regulation of the Nrf2/ARE pathway. NADPH oxidase, as a critical regulator of innate immunity, also limits lung inflammation by attenuating NF-κB and by activating Nrf2 [28,29]. On the other hand, CS activates the NF-κB pathway, which participates in the negative regulation of Nrf2/ARE signaling [30,31]. In addition, protein carbonylation induced by CS is involved in the suppression of the Nrf2/ARE pathway [32]. Because several studies have demonstrated that an Nrf2-dependent adaptive response is important in preventing CS-induced lung inflammation and injury [15,33,34], we reasoned that *Nrf2 *polymorphisms have a genetic impact on the CS-induced deterioration of lung function.

In the present study, we showed that a variant of the *Nrf2 *gene was associated with accelerated decline in FEV_1 _in a general population sample including non-smokers, moderate smokers, and heavy smokers. A stronger effect of the rs6726395 SNP on annual FEV_1 _decline was observed in smokers than in never-smokers, indicating a gene-smoking interaction in FEV_1 _decline. Such an interaction is reasonable because Nrf2 activation protects tissues against oxidative stress. The minor allele of rs6726395 in the homozygous state (A/A) was associated with a smaller FEV_1 _decline; therefore, this allele was thought to be protective against FEV_1 _decline.

The mechanisms mediating the relationship between rs6726395 and FEV_1 _decline were not determined in this study. Although the rs6726395 SNP is located in the first intron of the *Nrf2 *gene, rs6726395 variants did not correlate with different *Nrf2 *mRNA levels according to the GENEVAR database. However, given that GENEVAR utilizes only three cell types (fibroblast, lymphoblastoid cell line and T-cell), the possibility remains that rs6726395 has some genetic influence on *Nrf2* transcriptional activity in alveolar macrophages because Nrf2 appears to exert its protective effects through the transcriptional activation of antiprotease and antioxidant genes in alveolar macrophages [14] and its mRNA expression is decreased in macrophages of COPD patients [15]. As the rs6726395 SNP was not in significant LD with any of the 3 functional SNPs known to reside in the promoter region, rs6726395 may be in LD with other causal SNPs in or nearby the *Nrf2 *gene; the allele responsible for the protective effects observed in this study could be on the extended rs2001350T/rs6726395A/rs1962142A/rs2364722A/rs6721961T haplotype. We have not comprehensively assessed the genetic variation in *Nrf2*, and the functional impact of the *Nrf2 *SNPs carried on different haplotypes is still unknown. The identification of functional variants in *Nrf2 *loci will require fine mapping efforts using large populations. However, the lack of suitable Japanese cohorts with the measurements of annual decline in FEV_1 _and genomic DNA samples available for genotyping prevented us from performing a replication of this study on the association of rs6726395 with FEV_1 _decline.

In the present study, the annual declines in FEV_1 _were estimated by longitudinal retrospective measurements. The natural course of FEV_1 _over time is divided into three phases; a lung growth phase occurs during childhood and adolescence, this grow phase is followed by a plateau phase, and a decline phase begins at about 25 years of age [35]. The level of FEV_1 _at a given time in adulthood is affected by any deterioration that occurred in any of these 3 phases. Because we planned to analyze the effects of oxidative stress caused by cigarette smoking on lung function, FEV_1 _decline over time calculated in a longitudinal study was more valuable than absolute values of pulmonary function measurements in a cross-sectional study. As the ages of all the participants in this study were over 25 years at the first visit, all the subjects were thought to be in the decline phase of lung function.

Siedlinski et al. [17] have reported that the heterozygote genotype of rs2364723, which is in the first intron of *Nrf2*, is associated with a lower level of FEV_1 _in Caucasian smokers. However, they showed no relationship between rs2364723 and annual decline in FEV_1_. Because rs2364723 is in complete LD with rs2364722 (r^2 ^= 1.00) in the JPT population of the HapMap group, the finding from the Siedlinski et al. investigation may be compatible with the result from the present study. Our results are also consistent with findings from a previous Japanese case-control association study that showed no relationship between the 3 previously identified functional SNPs in the promoter region (rs6721961, rs6706649, and rs35652124) and susceptibility to COPD [36]. Recently, two large-scale genome-wide association studies have identified several loci associated with FEV_1 _and FEV_1_/FVC ratio [10,11]. Reports from the studies included lists of the top 2,000 SNPs related to the pulmonary function measurements. SNPs in or nearby the *Nrf2 *gene including the SNPs in the current study were not among these top 2,000 SNPs.

In order to ensure a reliable estimate of FEV_1 _decline, we selected the subjects who provided at least 4 valid PFT measurements over a period of at least 4 years. Variation in the follow-up periods and the numbers of visits could contribute to bias in estimations of annual FEV_1 _decline; therefore, we used a linear mixed-effects model to control for correlations among repeated measures from each subject. Moreover, inhaled corticosteroids, leukotriene receptor antagonists, and bronchodilators (e.g., β-adrenoceptor stimulants and anticholinergic agents) can improve FEV_1 _measurements in asthmatic and/or COPD subjects; therefore, we excluded those subjects with a history of asthma and/or COPD treated with these medications during the retrospective study period. Nevertheless, because this study was retrospective, attrition could affect the estimation of FEV_1 _decline. Subjects with accelerated decline in FEV_1 _are more likely to drop out from the annual health checkup. However, as attrition is likely to result in an underestimate of annual FEV_1 _decline, it would bias the study against finding an effect.

## Conclusions

We demonstrated an association between a SNP (rs6726395) in the first intron of the *Nrf2 *gene and annual decline in FEV_1_. In smokers, individuals carrying the major allele of this SNP showed greater decline in FEV_1 _than those homozygous for the minor allele; however, this effect was not seen in never-smokers. Although the direct functional effect of rs6726395 on the *Nrf2 *gene is unknown, this risk allele may be useful as a clinical marker for identifying individuals particularly susceptible to loss of lung function due to cigarette smoking. Our study suggests that pharmacological activation of Nrf2 by chemopreventive and phytochemical agents may be the strategy capable of exerting protective effects against various stress conditions including increased annual decline in FEV_1_ associated with CS.

## Competing interests

The authors declare that they have no competing interests.

## Authors' contributions

HM performed the subject recruitment, data collection, laboratory work, statistical analysis and manuscript writing. TN provided support in the development of the population. TS, HI and YK supervised clinical characterization and contributed to patient recruitment, the statistical analyses and the interpretation of clinical and genetic data. TH contributed to developing and performing the genotyping assays. MT contributed to overseeing the genotyping assays. EN contributed to overseeing the statistical methods and analysis of the genetic data. NH conceived the project design, supervised the study, discussed the results and finalized the manuscript. All authors contributed to and approved the final manuscript.

## Pre-publication history

The pre-publication history for this paper can be accessed here:

http://www.biomedcentral.com/1471-2350/12/97/prepub

## References

[B1] Lebowitz MD Quackenboss J Camilli AE Bronnimann D Holberg CJ Boyer B The epidemiological importance of intraindividual changes in objective pulmonary responses Eur J Epidemiol 1987 3 390 398 10.1007/BF00145651 3319671

[B2] Ulrik CS Lange P Decline of lung function in adults with bronchial asthma Am J Respir Crit Care Med 1994 150 629 634 8087330 10.1164/ajrccm.150.3.8087330

[B3] Wise RA The value of forced expiratory volume in 1 second decline in the assessment of chronic obstructive pulmonary disease progression Am J Med 2006 119 Suppl 1 4 11 16996894 10.1016/j.amjmed.2006.08.002

[B4] Fletcher CM Peto R Tinker CM Speizer FE The natural history of chronic bronchitis and emphysema. An eight-year study of early chronic obstructive lung disease in working men in London 1976 Oxford: Oxford University Press

[B5] Lange P Groth S Nyboe GJ Mortensen J Appleyard M Jensen G Schnohr P Effects of smoking and changes in smoking habits on the decline of FEV1 Eur Respir J 1989 2 811 816 2806504

[B6] Anthonisen NR Connett JE Kiley JP Altose MD Bailey WC Buist AS Conway WA Jr Enright PL Kanner RE O'Hara P Owens GR Scanlon PD Tashkin DP Wise RA Altose MD Connors AF Redline S Deitz C Rakos RF Conway WA Jr DeHorn A Ward JC Hoppe-Ryan CS Jentons RL Reddick JA Sawicki C Wise RA Permutt S Rand CS Scanlon PD Davis LJ Hurt RD Miller RD Williams DE Caron GM Lauger GG Toogood SM Buist AS Bjornson WM Johnson LR Bailey WC Brooks CM Dolce JJ Higgins DM Johnson MA Lorish CD Martin BA Tashkin DP Coulson AH Gong H Harber PI Li VC Roth M Nides MA Simmons MS Zuniga I Anthonisen NR Manfreda J Murray RP Rempel-Rossum SC Stoyko JM. Connett JE Kjelsberg MO Cowles MK Durkin DA Enright PL Kurnow KJ Lee WW Lindgren PG Mongin SJ O'Hara P Voelker HT Waller LA Owens GR Rogers RM Johnston JJ Pope FP Vitale FM Kanner RE Rigdon MA Benton KC Grant PM Becklake M Burrows B Cleary P Kimbel P Nett L Ockene JK Senior RM Snider GL Spitzer W Williams OD Hurd SS Kiley JP Wu MC Ayres SM Hyatt RE Mason BA Effects of smoking intervention and the use of an inhaled anticholinergic bronchodilator on the rate of decline of FEV1. The Lung Health Study JAMA 1994 272 1497 1505 10.1001/jama.272.19.1497 7966841

[B7] Pryor WA Biological effects of cigarette smoke, wood smoke, and the smoke from plastics: the use of electron spin resonance Free Radic Biol Med 1992 13 659 676 10.1016/0891-5849(92)90040-N 1334034

[B8] MacNee W Oxidants/antioxidants and COPD Chest 2000 117 Suppl 1 303S 317S 10843965 10.1378/chest.117.5_suppl_1.303s-a

[B9] Palmer LJ Knuiman MW Divitini ML Burton PR James AL Bartholomew HC Ryan G Musk AW Familial aggregation and heritability of adult lung function: results from the Busselton Health Study Eur Respir J 2001 17 696 702 10.1183/09031936.01.17406960 11401066

[B10] Repapi E Sayers I Wain LV Burton PR Johnson T Obeidat M Zhao JH Ramasamy A Zhai G Vitart V Huffman JE Igl W Albrecht E Deloukas P Henderson J Granell R McArdle WL Rudnicka AR Wellcome Trust Case Control Consortium Barroso I Loos RJ Wareham NJ Mustelin L Rantanen T Surakka I Imboden M Wichmann HE Grkovic I Jankovic S Zgaga L Hartikainen AL Peltonen L Gyllensten U Johansson A Zaboli G Campbell H Wild SH Wilson JF Gläser S Homuth G Völzke H Mangino M Soranzo N Spector TD Polasek O Rudan I Wright AF Heliövaara M Ripatti S Pouta A Naluai AT Olin AC Torén K Cooper MN James AL Palmer LJ Hingorani AD Wannamethee SG Whincup PH Smith GD Ebrahim S McKeever TM Pavord ID MacLeod AK Morris AD Porteous DJ Cooper C Dennison E Shaheen S Karrasch S Schnabel E Schulz H Grallert H Bouatia-Naji N Delplanque J Froguel P Blakey JD NSHD Respiratory Study Team Britton JR Morris RW Holloway JW Lawlor DA Hui J Nyberg F Jarvelin MR Jackson C Kähönen M Kaprio J Probst-Hensch NM Koch B Hayward C Evans DM Elliott P Strachan DP Hall IP Tobin MD Genome-wide association study identifies five loci associated with lung function Nat Genet 2010 42 36 44 10.1038/ng.501 20010834 PMC2862965

[B11] Hancock DB Eijgelsheim M Wilk JB Gharib SA Loehr LR Marciante KD Franceschini N van Durme YM Chen TH Barr RG Schabath MB Couper DJ Brusselle GG Psaty BM van Duijn CM Rotter JI Uitterlinden AG Hofman A Punjabi NM Rivadeneira F Morrison AC Enright PL North KE Heckbert SR Lumley T Stricker BH O'Connor GT London SJ Meta-analyses of genome-wide association studies identify multiple loci associated with pulmonary function Nat Genet 2010 42 45 52 10.1038/ng.500 20010835 PMC2832852

[B12] Kobayashi M Yamamoto M Nrf2-Keap1 regulation of cellular defense mechanisms against electrophiles and reactive oxygen species Adv Enzyme Regul 2006 46 113 140 10.1016/j.advenzreg.2006.01.007 16887173

[B13] Itoh K Chiba T Takahashi S Ishii T Igarashi K Katoh Y Oyake T Hayashi N Satoh K Hatayama I Yamamoto M Nabeshima Y An Nrf2/small Maf heterodimer mediates the induction of phase II detoxifying enzyme genes through antioxidant response elements Biochem Biophys Res Commun 1997 236 313 322 10.1006/bbrc.1997.6943 9240432

[B14] Ishii Y Itoh K Morishima Y Kimura T Kiwamoto T Iizuka T Hegab AE Hosoya T Nomura A Sakamoto T Yamamoto M Sekizawa K Transcription factor Nrf2 plays a pivotal role in protection against elastase-induced pulmonary inflammation and emphysema J Immunol 2005 175 6968 6975 16272357 10.4049/jimmunol.175.10.6968

[B15] Suzuki M Betsuyaku T Ito Y Nagai K Nasuhara Y Kaga K Kondo S Nishimura M Down-regulated NF-E2-related factor 2 in pulmonary macrophages of aged smokers and patients with chronic obstructive pulmonary disease Am J Respir Cell Mol Biol 2008 39 673 682 10.1165/rcmb.2007-0424OC 18566336

[B16] Marzec JM Christie JD Reddy SP Jedlicka AE Vuong H Lanken PN Aplenc R Yamamoto T Yamamoto M Cho HY Kleeberger SR Functional polymorphisms in the transcription factor NRF2 in humans increase the risk of acute lung injury FASEB J 2007 21 2237 2246 10.1096/fj.06-7759com 17384144

[B17] Siedlinski M Postma DS Boer JM van der Steege G Schouten JP Smit HA Boezen HM Level and course of FEV1 in relation to polymorphisms in NFE2L2 and KEAP1 in the general population Respir Res 2009 10 73 10.1186/1465-9921-10-73 19671143 PMC2738671

[B18] Masuko H Sakamoto T Kaneko Y Iijima H Naito T Noguchi E Hirota T Tamari M Hizawa N Lower FEV1 in non-COPD, non-asthmatic subjects: association with smoking, annual decline in FEV1, total IgE levels, and TSLP genotypes Int J Chron Obstruct Pulmon Dis 2011 6 181 189 21468164 10.2147/COPD.S16383 PMC3064418

[B19] The Committee of Pulmonary Physiology, Japanese Respiratory Society Guideline of respiratory function tests. Spirometry, flow-volume curve, diffusion capacity of the lung Nihon Kokyuki Gakkai Zasshi 2004 Suppl 1 56 (in Japanese) 15565748

[B20] de Bakker PIW Yelensky R Pe'er I Gabriel SB Daly MJ Altshuler D Efficiency and power in genetic association studies Nat Genet 2005 37 1217 1223 10.1038/ng1669 16244653

[B21] Barrett JC Fry B Maller J Daly MJ Haploview: analysis and visualization of LD and haplotype maps Bioinformatics 2005 21 263 265 10.1093/bioinformatics/bth457 15297300

[B22] Hill WG Robertson A The effect of linkage on limits to artificial selection Genet Res 1966 8 269 294 10.1017/S0016672300010156 5980116

[B23] Yang TP Beazley C Montgomery SB Dimas AS Gutierrez-Arcelus M Stranger BE Deloukas P Dermitzakis ET Genevar: a database and Java application for the analysis and visualization of SNP-gene associations in eQTL studies Bioinformatics 2010 26 2474 2476 10.1093/bioinformatics/btq452 20702402 PMC2944204

[B24] Dimas AS Deutsch S Stranger BE Montgomery SB Borel C Attar-Cohen H Ingle C Beazley C Gutierrez Arcelus M Sekowska M Gagnebin M Nisbett J Deloukas P Dermitzakis ET Antonarakis SE Common regulatory variation impacts gene expression in a cell type-dependent manner Science 2009 325 1246 1250 10.1126/science.1174148 19644074 PMC2867218

[B25] Schaid DJ Rowland CM Tines DE Jacobson RM Poland GA Score tests for association between traits and haplotypes when linkage phase is ambiguous Am J Hum Genet 2002 70 425 434 10.1086/338688 11791212 PMC384917

[B26] Nagai K Betsuyaku T Kondo T Nasuhara Y Nishimura M Long term smoking with age builds up excessive oxidative stress in bronchoalveolar lavage fluid Thorax 2006 61 496 502 10.1136/thx.2005.049148 16537669 PMC2111210

[B27] Rytilä P Rehn T Ilumets H Rouhos A Sovijärvi A Myllärniemi M Kinnula VL Increased oxidative stress in asymptomatic current chronic smokers and GOLD stage 0 COPD Respir Res 2006 7 69 10.1186/1465-9921-7-69 16646959 PMC1524947

[B28] Yao H Edirisinghe I Yang SR Rajendrasozhan S Kode A Caito S Adenuga D Rahman I Genetic ablation of NADPH oxidase enhances susceptibility to cigarette smoke-induced lung inflammation and emphysema in mice Am J Pathol 2008 172 1222 1237 10.2353/ajpath.2008.070765 18403597 PMC2329832

[B29] Segal BH Han W Bushey JJ Joo M Bhatti Z Feminella J Dennis CG Vethanayagam RR Yull FE Capitano M Wallace PK Minderman H Christman JW Sporn MB Chan J Vinh DC Holland SM Romani LR Gaffen SL Freeman ML Blackwell TS NADPH oxidase limits innate immune responses in the lungs in mice PLoS One 2010 5 e9631 10.1371/journal.pone.0009631 20300512 PMC2838778

[B30] Yang SR Chida AS Bauter MR Shafiq N Seweryniak K Maggirwar SB Kilty I Rahman I Cigarette smoke induces proinflammatory cytokine release by activation of NF-kappaB and posttranslational modifications of histone deacetylase in macrophages Am J Physiol Lung Cell Mol Physiol 2006 291 L46 57 10.1152/ajplung.00241.2005 16473865

[B31] Garbin U Fratta Pasini A Stranieri C Cominacini M Pasini A Manfro S Lugoboni F Mozzini C Guidi G Faccini G Cominacini L Cigarette smoking blocks the protective expression of Nrf2/ARE pathway in peripheral mononuclear cells of young heavy smokers favouring inflammation PLoS One 2009 4 e8225 10.1371/journal.pone.0008225 20011043 PMC2784946

[B32] Kode A Rajendrasozhan S Caito S Yang SR Megson IL Rahman I Resveratrol induces glutathione synthesis by activation of Nrf2 and protects against cigarette smoke-mediated oxidative stress in human lung epithelial cells Am J Physiol Lung Cell Mol Physiol 2008 294 L478 488 18162601 10.1152/ajplung.00361.2007

[B33] Iizuka T Ishii Y Itoh K Kiwamoto T Kimura T Matsuno Y Morishima Y Hegab AE Homma S Nomura A Sakamoto T Shimura M Yoshida A Yamamoto M Sekizawa K Nrf2-deficient mice are highly susceptible to cigarette smoke-induced emphysema Genes Cells 2005 10 1113 1125 10.1111/j.1365-2443.2005.00905.x 16324149

[B34] Blake DJ Singh A Kombairaju P Malhotra D Mariani TJ Tuder RM Gabrielson E Biswal S Deletion of Keap1 in the lung attenuates acute cigarette smoke-induced oxidative stress and inflammation Am J Respir Cell Mol Biol 2010 42 524 536 10.1165/rcmb.2009-0054OC 19520915 PMC2874439

[B35] Kerstjens HA Rijcken B Schouten JP Postma DS Decline of FEV1 by age and smoking status: facts, figures, and fallacies Thorax 1997 52 820 827 10.1136/thx.52.9.820 9371217 PMC1758654

[B36] Yamamoto T Yoh K Kobayashi A Ishii Y Kure S Koyama A Sakamoto T Sekizawa K Motohashi H Yamamoto M Identification of polymorphisms in the promoter region of the human NRF2 gene Biochem Biophys Res Commun 2004 321 72 79 10.1016/j.bbrc.2004.06.112 15358217

